# Excited States Symmetry Breaking and In-Plane Polarization Cause Chiral Reversal in Diastereomers

**DOI:** 10.3390/molecules26154680

**Published:** 2021-08-02

**Authors:** Chenglong Wang, Jingang Wang, Chunyang Wang

**Affiliations:** 1 School of Electronic Information Engineering, Changchun University of Science and Technology, Changchun 130022, China; 13944934139@139.com; 2 Changchun Institute of Optics, Fine Mechanics and Physics, Chinese Academy of Sciences, Changchun 130033, China; 3 College of Science, Liaoning Petrochemical University, Fushun 113001, China; 4 Northwest Institutes of Advanced Technology, Xi’an Technological University, Xi’an 710021, China

**Keywords:** electronic transitions, chirality reversal, electromagnetic interaction

## Abstract

In this work, we investigate the electronic transitions and chirality of three isomers of huge conjugated systems: asymmetric diastereomers (MMMM) and two symmetrical diastereomers (PMPM and PPMM). The physical mechanism of flipping has been studied theoretically. The new ribbon-shaped polycyclic aromatic hydrocarbons (PAHs) molecule is formed by connecting three graphene-like systems with large conjugated π orbitals. By calculating and analyzing electromagnetic interaction decomposition over distance, it can be found that the chirality reversal of different energies is caused by the symmetrical fracture of TMDM in the Z direction. The chirality reversal at the same energy is caused by the in-plane polarization of the TMDM along the Y direction.

## 1. Introduction

Chirality is a phenomenon in which the real object and its mirror image do not overlap. This phenomenon is common in nature, ranging from molecules and cells on the microscopic level to large galaxies in the universe [1,2,3,4]. The concept of chirality was first discovered in 1808 when French physicist Malus discovered the polarized light phenomenon of calcite. Then, in 1815, Jon Baptiste Biot discovered that polarized light is reflected at a certain angle when passing through certain organic solutions [5], because the organic solute has a certain optical activity. In 1848, Pasteur discovered that gluconate crystals with different growth directions have opposite polarized light effects [6]. In 1874, Dutch scientist Uan’t Hoff and French scientist Le Bel proposed the Stereo conformation theory of molecules, which provides a more reasonable explanation for the phenomenon of the same molecular formula but inconsistent optical activity [7].

Chiral phenomena can also explain the origin of life [8], there are a large number of chiral molecules in life systems, such as amino acids, proteins, nucleic acids and ribonucleic acids, which are almost composed of basic entities with a single optical activity and play a key role in life activities [9]. In chiral research, chiral molecules with a single optical activity are used by optical chirality characterization, chiral transport of charges, etc., in asymmetric synthesis [10,11], pesticides [12], medicine [13], enantiomeric separation [14], optoelectronic devices [15], catalysis [16] and other fields. The medicinal mechanism of pesticides and medicines acting on organisms is mainly related to their chiral matching with target molecules in the body [17]. The use of chiral targeting can realize molecular recognition and separation [18,19]. In addition, chiral catalysis is an effective method of catalysis, which induces a large number of chiral molecular products through catalytic molecules. A large number of chiral molecule products with excellent characteristics are also generated through the water phase [20], fluorine phase [21], ionic liquid [22], supercritical carbon dioxide [23] and other heterogeneous chiral catalytic means; in the pharmaceutical industry; and in materials science and agriculture, which greatly improves the catalytic efficiency.

**Figure 1:**
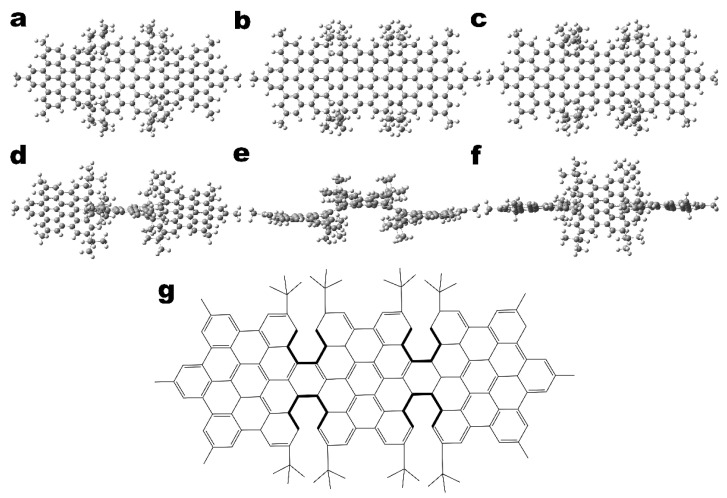
The molecular structure of the three isomers. (**a**) The molecular structure diagram of the asymmetric isomer MMMM, PPMM and PMPM (**a**–**c**), and their side view (**d**–**f**) and chemical structure (**g**).

The phenomenon of isomerism widely exists in organic chemistry research, which is one of the reasons for the huge number of organic compounds. Isoisomerism is divided into structural isomerism and stereoisomerism, and enantiomerism is a kind of stereoisomerism related to the optical properties of substances and belongs to optical isomerism. In organic chemistry, molecules with enantiomers (the real object and the mirror image cannot overlap after translation) are called chiral molecules. Chiral molecules are generally optically active, that is, when a beam of plane-polarized light irradiates the chiral compound molecule solution, the direction of the polarization plane of the emitted light rotates by an angle [24,25,26]. The molecules studied in this paper are diastereoisomers with optical rotation, as shown in Figure 1. Diastereoisomers are a new type of ribbon polycyclic aromatic hydrocarbons (PAHs) fused into an aromatic skeleton by three HBC-type scaffolds, in which hexa-peri-hexabenzocoronene (HBC) can provide the required large conjugated π surface suitable for multiphoton absorption capacity. Moreover, this is also a substrate for the synthesis of graphene nanoribbons, so it can be called ribbon-like. The aromatic skeleton has high solubility in organic solvents [27], and the novel ribbon-shaped PAHs with three different molecular configurations provide potential applications for molecular chirality. In this work, electronic circular dichroism (ECD) can effectively characterize the chirality of the chromophore in the molecule, and the chirality of the same molecule can be reflected in the ECD spectrum. Besides, the tensor product of the transition electric dipole moments (TEDMs) and the transition magnetic dipole moments (TMDMs) is visualized to analyze the physical mechanism of symmetry changing the molecular chirality. The physical mechanism of this chiral optical reversal may be different from the traditional reversal of the chiral center, but the asymmetric magnetic transition is caused by the overall distortion of the chromophore. The generalized theoretical calculation research on the physical mechanism of chirality caused by the structural twist of PAHs is very meaningful for understanding the source of chirality in bilayer graphene and twisted bilayer graphene (TBG).

## 2. Method

### 2.1. Computational Details

The ribbon-shaped PAHs molecules in three configurations (Figure 1) were used as target molecules for studying the optical properties of ultraviolet-visible (UV-Vis) and ECD. In the preliminary work, the UV-Vis spectrum was studied experimentally. However, the physical mechanism by which symmetry changes the chirality of molecules is unclear. In this paper, theoretical calculations by Gaussian 16 (Gaussian Inc., Wallingford, CT, USA) were carried out to fully reveal the changes of molecular chirality with molecular configuration or conformation [28]. Using the time-dependent density functional theory (TDDFT) [29], CAM-B3LYP functional [30] and 6–31 + G (d) basis set [31], Gaussian calculation was performed on the molecules, as described by the authors of [27]. This method is very suitable for calculating molecular excitation, and the calculation of the wave function is more accurate, thereby obtaining more accurate UV-Vis spectrum and ECD spectrum. Multiwfn 3.6 (Beijing Kein Research Center for Natural Sciences, Beijing, China) was used to deal with the transition electric/magnetic dipole moment, transition electric/magnetic dipole moment density matrix and electron-hole pair analysis [32]. Two- and three-dimensional graphs were drawn by VMD 1.9 software (Urbana, IL, USA) [33].

### 2.2. ECD

The molecular chirality comes from the anisotropy of the transition electric dipole moment and the transition magnetic dipole moment, which can be well observed with ECD. The ECD intensity is [34,35]:

(1) $$I\propto {\left|\langle \varphi_{j}|\mu_{e}|\varphi_{i}\rangle E\right|}^{2}+{\left|\langle \varphi_{j}|\mu_{e}|\varphi_{i}\rangle \langle \varphi_{j}|\mu_{m}|\varphi_{i}\rangle B\right|}^{2}$$

where the first term in the formula is light absorption, and the second term represents the tensor product of the transition electromagnetic dipole moment as ECD, where $\mu_{e}$ and $\mu_{m}$ are the transition electric dipole moment and transition magnetic dipole moment, respectively.

### 2.3. TEDMs and TMDMs

The matrix element of TEDMs and TMDMs is defined as the dipole moment product between the corresponding transition dipole moment (TDM) matrix element and the corresponding basis function. TEDMs and TMDMs have three components: X, Y and Z (such as the sum of all matrix elements of the electric X component matrix is exactly the X component of the system TEDMs). It is also possible to shrink the TEDMs/TMDMs matrix into an atom-based form. The X, Y and Z components of the atomic TEDMs/TMDMs matrix are defined as [35]:

(2) $$\{\begin{array}{c} D_{A,B}^{X}=\sum_{\mu \in A}\sum_{\nu \in B}p_{\mu \nu }^{tran}\left[\langle \chi_{\mu }|-x|\chi_{\mu }\rangle +\frac{\sum_{\mu \neq \nu }\left(\langle \chi_{\mu }|-x|\chi_{\nu }\rangle +\langle \chi_{\nu }|-x|\chi_{\mu }\rangle \right)}{2}\right]\\ D_{A,B}^{Y}=\sum_{\mu \in A}\sum_{\nu \in B}p_{\mu \nu }^{tran}\left[\langle \chi_{\mu }|-y|\chi_{\mu }\rangle +\frac{\sum_{\mu \neq \nu }\left(\langle \chi_{\mu }|-y|\chi_{\nu }\rangle +\langle \chi_{\nu }|-y|\chi_{\mu }\rangle \right)}{2}\right]\\ D_{A,B}^{Z}=\sum_{\mu \in A}\sum_{\nu \in B}p_{\mu \nu }^{tran}\left[\langle \chi_{\mu }|-z|\chi_{\mu }\rangle +\frac{\sum_{\mu \neq \nu }\left(\langle \chi_{\mu }|-z|\chi_{\nu }\rangle +\langle \chi_{\nu }|-z|\chi_{\mu }\rangle \right)}{2}\right] \end{array}$$

(3) $$\{\begin{array}{c} M_{A,B}^{X}=\sum_{\mu \in A}\sum_{\nu \in B}p_{\mu \nu }^{tran}\left[\begin{array}{l} \langle \chi_{\mu }|x\frac{d}{dy}-y\frac{d}{dx}|\chi_{\mu }\rangle \\ +\frac{\sum_{\mu \neq \nu }\left(\langle \chi_{\mu }|x\frac{d}{dy}-y\frac{d}{dx}|\chi_{\nu }\rangle +\langle \chi_{\nu }|x\frac{d}{dy}-y\frac{d}{dx}|\chi_{\mu }\rangle \right)}{2} \end{array}\right]\\ M_{A,B}^{Y}=\sum_{\mu \in A}\sum_{\nu \in B}p_{\mu \nu }^{tran}\left[\begin{array}{l} \langle \chi_{\mu }|y\frac{d}{dz}-z\frac{d}{dy}|\chi_{\mu }\rangle \\ +\frac{\sum_{\mu \neq \nu }\left(\langle \chi_{\mu }|y\frac{d}{dz}-z\frac{d}{dy}|\chi_{\nu }\rangle +\langle \chi_{\nu }|y\frac{d}{dz}-z\frac{d}{dy}|\chi_{\mu }\rangle \right)}{2} \end{array}\right]\\ M_{A,B}^{Z}=\sum_{\mu \in A}\sum_{\nu \in B}p_{\mu \nu }^{tran}\left[\begin{array}{l} \langle \chi_{\mu }|z\frac{d}{dx}-x\frac{d}{dz}|\chi_{\mu }\rangle \\ +\frac{\sum_{\mu \neq \nu }\left(\langle \chi_{\mu }|z\frac{d}{dx}-x\frac{d}{dz}|\chi_{\nu }\rangle +\langle \chi_{\nu }|z\frac{d}{dx}-x\frac{d}{dz}|\chi_{\mu }\rangle \right)}{2} \end{array}\right] \end{array}$$

where μ and ν are basis functions in atoms *A* and *B*, $\chi_{\mu }$ represents the amount of basis function μ, and $p_{\mu \nu }^{tran}$ is the TDM, which can be expressed by the following formula:

(4) $$p_{\mu \nu }^{tran}=\sum_{i}^{occ}\sum_{j}^{vir}w_{i}^{j}C_{\mu i}C_{\nu j}$$

where i and j represent the empty orbit and occupied orbit. The expansion coefficient of function μ on the orbit i is denoted by $C_{ui}$, and ω is the distribution coefficient from the occupied orbit j to the empty orbit.

## 3. Results and Discussion

### 3.1. UV-Vis Absorption Spectra

**Figure 2:**
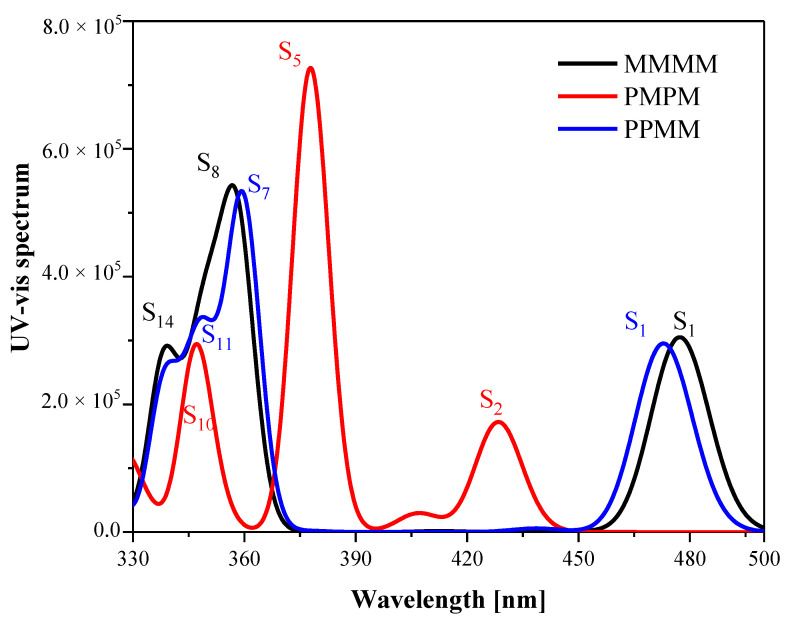
The UV-Vis spectra of three isomeric molecules. The black line represents the UV-Vis spectrum of the asymmetric isomer (The S1, S8, S14 of MMMM), the red line represents the UV-Vis spectrum of the symmetric diastereomer (The S2, S5, S10 of PMPM) and the blue line represents the UV-Vis spectrum of the symmetric diastereomer (The S1, S7, S11 of PPMM).

The UV-Vis spectra of the three isomers of new ribbon PAHs molecules are shown in Figure 2. The contribution of the excited state in the figure comes from the linear combination of atomic energy levels, which is the subspace invariance required in the TDDFT principle. Theoretical calculations provide all possible excited states and their intensities, but the weaker excited states (the oscillator strength smaller than 0.0001) may not be observed experimentally. Strong peaks in the spectrum between 330–390 nm are contributed by the excitation states of S8 (MMMM, 357.24 nm), S5 (PMPM, 377.57 nm) and S7 (PPMM, 359.96 nm), respectively. Two symmetric diastereomers (PMPM and PPMM) are red-shifted relative to the asymmetric isomer (MMMM) spectrum, respectively, to the infrared direction, with shifts of 20.33 nm (PMPM) and 2.72 nm (PPMM). In addition, the peaks of the spectrum between 420 nm and 500 nm are contributed by the excitation states of S1 (MMMM, 476.99 nm), S2 (PMPM, 428.17 nm) and S1 (PPMM, 472.61 nm). The two symmetric diastereomers (PMPM and PPMM) have a blue shift relative to the spectrum of the asymmetric isomer (MMMM), with shifts of 48.82 nm (PMPM) and 4.38 nm (PPMM), respectively. Symmetry causes the UV-Vis spectra of PMPM and PPMM to shift relative to MMMM, where the PMPM spectrum shifts the most.

The TDM is one of the most critical quantities for discussing electronic excitation. Only when the transition dipole moment is large, the oscillator strength is large, and the corresponding absorption can be relatively strong. The new ribbon-shaped PAHs molecule was synthesized from three HBCs, and the electronic excitation characteristics were further studied by analyzing the TDM of different HBC fragments in the molecule. The contribution of the *r*-th basis function to the TDM is written as:

(5) $$d_{r}=\sum_{s}P_{r,s}^{tran}\langle \chi_{r}|-r|\chi_{s}\rangle$$

where $P_{r,s}^{tran}$ is the TDM between two certain states, and the latter term is the electric dipole moment integral between the basis functions of r and s. The basis function contributions are summed up by atoms, and the contribution of atoms to the transition dipole moment was obtained. The contribution of all basis functions in the segment was added to obtain the TDM of the segment.

**Figure 3:**
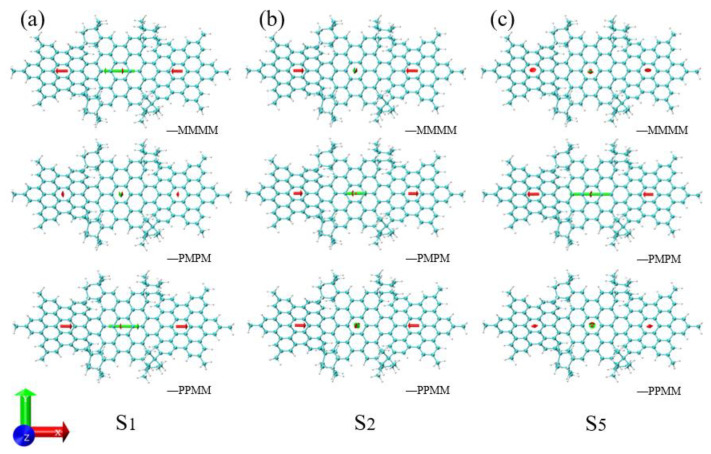
The TDM of S1, S2 and S5 of three isomeric molecules. (**a**) The TDM of three molecules S1, (**b**) the TDM of three molecules S2, (**c**) the TDM of three molecules S5. Red represents the TDM of the PAHs segment, green represents the total TDM of the molecule, the arrow represents the direction of the TDM and the length of the arrow represents the size of the TDM.

We performed TDM analysis on S1, S2 and S5 of the three molecules, and found that the total TDM (green) is mainly in the x direction, as shown in Figure 3. The length of the green arrows of MMMM and PPMM in Figure 3a is significantly greater than that of PMPM, and the length of the green arrows of PMPM is too small, so only the S1 peaks of MMMM and PPMM are displayed in the spectrum. In addition, the TDM of PPMM in Figure 3a is opposite to that of MMMM, and the length is slightly smaller than that of MMMM, so the spectrum of S1 of PPMM is slightly lower than that of MMMM. In Figure 3b,c, the length of the green arrow of PMPM is obviously greater than that of MMMM and PPMM, and the length of S5 is greater than that of S2, so the spectrum shows two peaks (S5—strong peak, S2—weak peak). By carefully observing the configuration diagrams of the three molecules (Figure 1), it can be found that symmetry changes the degree of distortion of the molecular structure of PMPM and PPMM relative to MMMM. This causes the TDM of the excited state to change, resulting the shift of UV-Vis spectrum.

**Figure 4:**
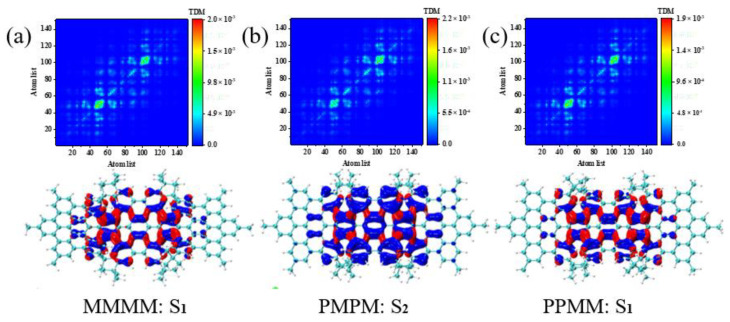
The TDM and CDD of the excited states of three isomeric molecules at 390–500 nm in the spectrum. (**a**) The TDM and CDD diagrams of S1 of MMMM at 477 nm. (**b**) The TDM and CDD diagrams of S2 of PMPM at 428 nm. (**c**) The TDM and CDD diagrams of S1 of PPMM at 472 nm.

**Figure 5:**
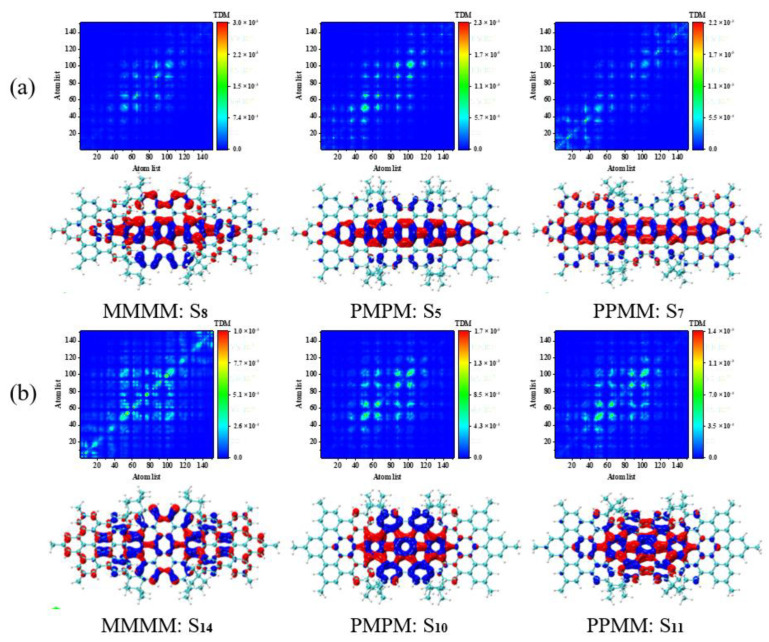
The TDM and CDD of three isomeric molecules’ excited states at 330–390 nm in the spectrum. (**a**) The TDM and CDD diagrams of the excited state corresponding to the strong peak are S8 of MMMM, S5 of PMPM and S7 of PPMM, respectively; (**b**) the TDM and CDD diagrams of the excited state corresponding to the sub-strong peak are S14 of MMMM, S10 of PMPM and S11 of PPMM, respectively.

We perform hole-electron pair analysis on the main excited states of 330–500 nm in the UV-Vis spectrum to better analyze the mechanism of intramolecular charge transfer, as shown in Figure 4 and Figure 5. In the charge density difference (CDD) figure, the blue isosurface represents holes, and the red isosurface represents electrons. The holes and electrons in the excited states of the three molecules have the characteristics of close distribution, high degree of overlap and no obvious separation, so they are integrally excited. In Figure 4, holes-electrons are concentrated on the three HBC connections and the middle HBC, and most of the electrons are concentrated on the benzene ring at the HBC connections. In Figure 5a, the holes-electrons of the asymmetric isomer (MMMM) are mainly distributed in the middle HBC, and the electrons are transferred from bottom to top at the middle HBC.

The difference is that the holes-electrons of the two symmetrical diastereomers (PMPM and PPMM) are mainly distributed on the central benzene chain in the long axis direction. In addition, the red isosurface appears on mostly both sides of the HBC, and the blue isosurface appears mostly in the central HBC. Therefore, electrons are transferred from the center to both sides. In Figure 5b, the asymmetric isomer (MMMM) holes are mainly distributed on the central benzene chain in the long axis direction. Thus, the electrons are transferred from the center to both sides. The difference is that the holes-electrons of the two symmetrical diastereomers (PMPM and PPMM) are mainly distributed in the middle HBC. The two symmetrical diastereomers (PMPM and PPMM) and the asymmetric isomer (MMMM) have exactly the opposite excitation regions under the corresponding peaks, so the symmetry changes the excitation positions of the three PAHs isomers.

**Figure 6:**
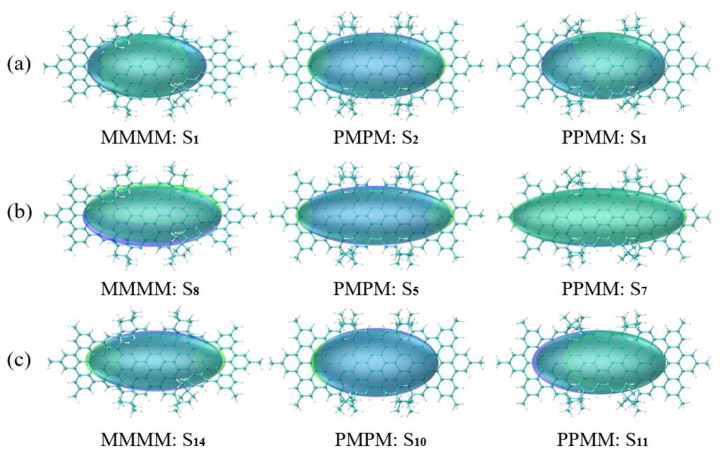
The hole-electron smooth distribution diagram of excited states of three isomeric molecules. The green isosurface represents electrons, and the blue isosurface represents holes. (**a**) The hole-electron smooth distribution diagram of the excited states of three isomeric molecules at 390–500 nm in the spectrum (There are S1 of MMMM at 477 nm, S2 of PMPM at 428 nm and S1 of PPMM at 472 nm, respectively); (**b**) The hole-electron smooth distribution diagram of the excited state corresponding to the strong peak are S8 of MMMM, S5 of PMPM and S7 of PPMM, respectively; (**c**) The hole-electron smooth distribution diagram of the excited state corresponding to the sub-strong peak are S14 of MMMM, S10 of PMPM and S11 of PPMM, respectively.

The distribution of holes and electrons in the excited state CDD of isomeric molecules is alternate and messy, which makes visualization research challenging. In this regard, we define Chole and Cele to describe the distribution of holes and electrons smoothly, as shown in Figure 6.

(6) $$\{\begin{array}{c} C_{ele}\left(r\right)=A_{ele}\exp\left(-\frac{{\left(x-X_{ele}\right)}^{2}}{2\sigma_{ele,x}^{2}}-\frac{{\left(y-Y_{ele}\right)}^{2}}{2\sigma_{ele,y}^{2}}-\frac{{\left(z-Z_{ele}\right)}^{2}}{2\sigma_{ele,z}^{2}}\right)\\ C_{hole}\left(r\right)=A_{hole}\exp\left(-\frac{{\left(x-X_{hole}\right)}^{2}}{2\sigma_{hole,x}^{2}}-\frac{{\left(y-Y_{hole}\right)}^{2}}{2\sigma_{hole,y}^{2}}-\frac{{\left(z-Z_{hole}\right)}^{2}}{2\sigma_{hole,z}^{2}}\right) \end{array}$$

where *A* is the normalization coefficient, which makes the whole space integral of $C_{ele}$ and $C_{hole}$ both equal to 1. x, y, z are the three Cartesian components of the coordinate vector *r*. *σ* is the overall distribution breadth of electrons in the x direction, which can be expressed by the following formula:

(7) $$\sigma_{ele,x}^{2}=\int {\left(x-X_{ele}\right)}^{2}\rho^{ele}\left(r\right)dr$$

$C_{hole}$ and $C_{ele}$ are equivalent to describing the distribution of holes and electrons with a Gaussian function to erase the distribution details. At this time, the isosurface diagram of holes and electrons becomes an elliptical shape, which is obviously clearer and more intuitive in graphical inspection. In Figure 6, the degree of overlap between $C_{hole}$ and $C_{ele}$ isosurfaces is large, and the distance between the center of mass of the hole and the electron is small, so the excited states are in overall excitation. In Figure 6a, the $C_{hole}$ or $C_{ele}$ isosurface is an oblate, which means that the extent of extension in the shortest axis direction is much lower than in other directions. In Figure 6b, the $C_{hole}$ or $C_{ele}$ isosurface looks like an apparent ellipse, which means that the degree of extension in the long axis direction is much higher than in other directions. In addition, the two symmetrical diastereomers (PMPM and PPMM) have a much higher degree of extension in the long axis direction than the asymmetric isomer (MMMM). Compared with Figure 6b, the asymmetric isomer (MMMM) in Figure 6c has an increased degree of extension in the long axis direction, the extension of the two symmetrical diastereomers (PMPM and PPMM) in the long axis direction is significantly reduced and the short axis direction is increased.

### 3.2. ECD Spectra

**Figure 7:**
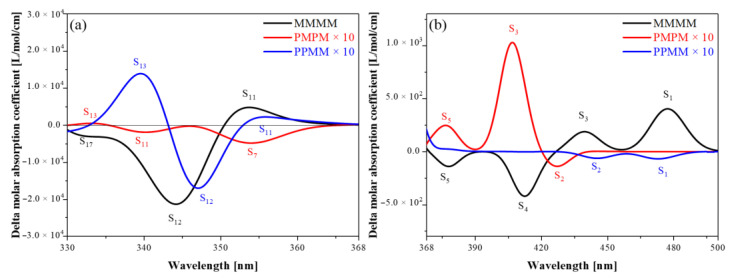
The ECD of three isomeric molecules. The black line represents the ECD of the asymmetric isomer (MMMM), the red line indicates the ECD of the symmetric diastereomer (PMPM) expanded by 10 times and the blue line represents the ECD of the symmetric diastereomer (PPMM) expanded by a factor of 10. (**a**) The ECD of three isomeric molecules at 330–368 nm. Where, the black peaks correspond to S11, S12, and S17 of MMMM, the red peaks correspond to S7, S11, and S13 of PMPM, and the blue peaks correspond to S11, S12, and S13 of PPMM, respectively; (**b**) the ECD of three isomeric molecules at 368–500 nm. Where, the black peaks correspond to S1, S3, S4 and S5 of MMMM, the red peaks correspond to S2, S3, and S5 of PMPM, and the blue peaks correspond to S1 and S2 of PPMM, respectively.

**Figure 8:**
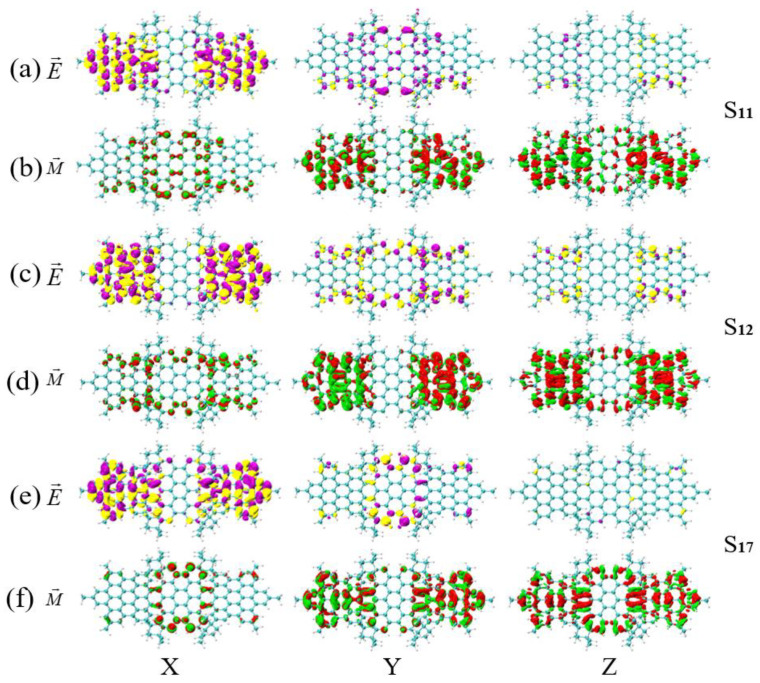
The TEDM and TMDM of S11, S12 and S17 of asymmetric isomers (MMMM). (**a**,**b**) are the TEDM and TMDM of S11 in the x/y/z direction; (**c**,**d**) are the TEDM and TMDM of S13 in the x/y/z direction; (**e**,**f**) are the TEDM and TMDM of S17 in the x/y/z direction. The letters E and M in the figure indicate the TEDM and TMDM of the molecule, respectively.

**Figure 9:**
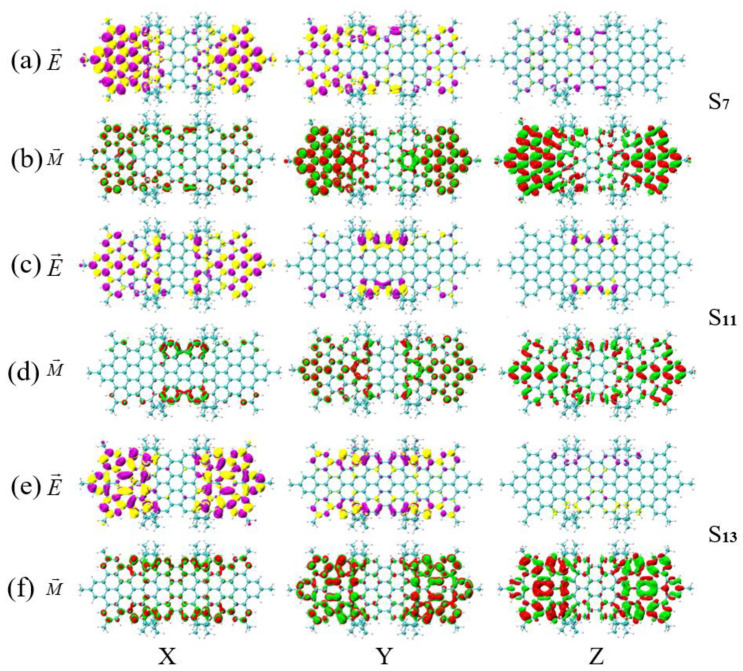
The TEDM and TMDM of S7, S11 and S13 of symmetrical diastereomers (PMPM). (**a**,**b**) are the TEDM and TMDM of S7 in the x/y/z direction; (**c**,**d**) are the TEDM and TMDM of S11 in the x/y/z direction; (**e**,**f**) are the TEDM and TMDM of S13 in the x/y/z direction. The letters E and M in the figure indicate the TEDM and TMDM of the molecule, respectively.

**Figure 10:**
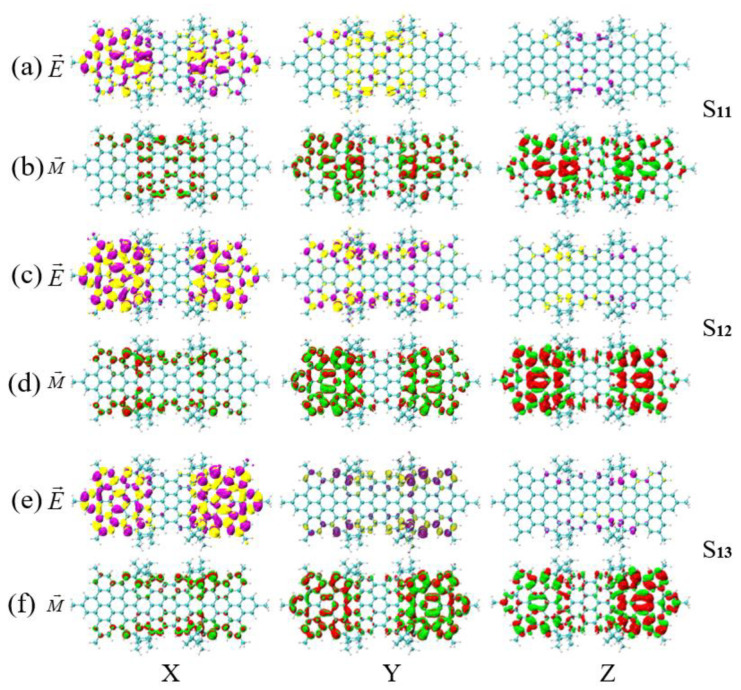
The TEDM and TMDM of S11, S12 and S13 of symmetrical diastereomers (PPMM). (**a**,**b**) are the TEDM and TMDM of S11 in the x/y/z direction; (**c**,**d**) are the TEDM and TMDM of S12 in the x/y/z direction; (**e**,**f**) are the TEDM and TMDM of S13 in the x/y/z direction. The letters E and M in the figure indicate the TEDM and TMDM of the molecule, respectively.

**Figure 11:**
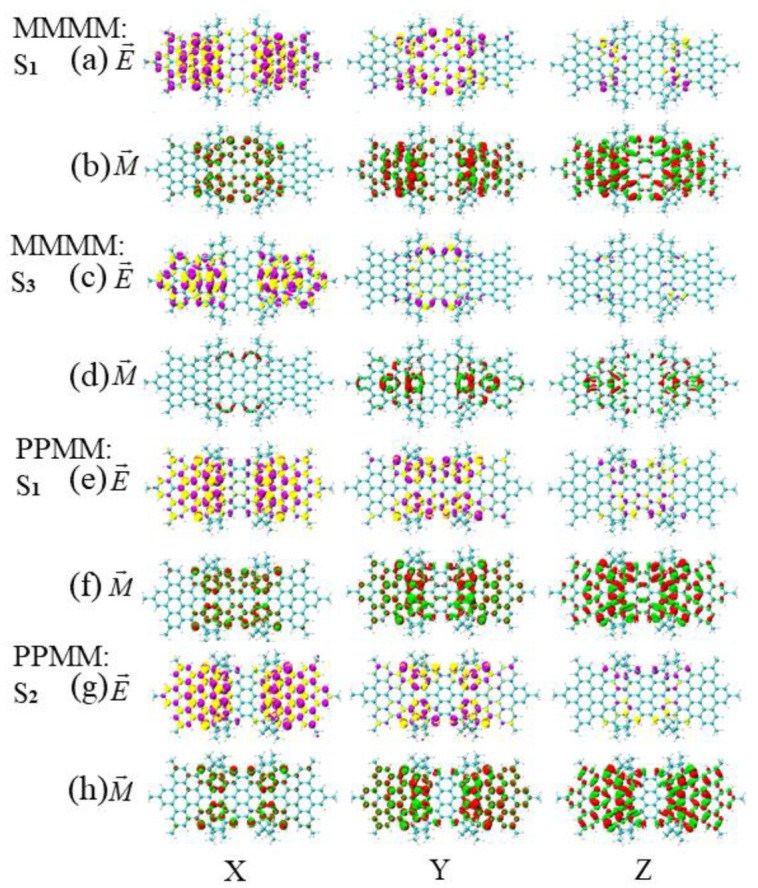
The TEDM and TMDM at similar wavelengths for asymmetric isomers (MMMM) and symmetric diastereomers (PPMM). (**a**,**b**) are distributed as the TEDM and TMDM in the x/y/z/direction of S1 of MMMM near the wavelength of 477 nm; (**c**,**d**) are distributed as the TEDM and TMDM in the x/y/z direction of S3 of MMMM near the wavelength of 438 nm; (**e**,**f**) are distributed as the TEDM and TMDM in the x/y/z direction of S1 of PPMM near the wavelength of 472 nm; (**g**,**h**) are distributed as the TEDM and TMDM in the x/y/z direction of S2 of PPMM near the wavelength of 444 nm. The letters E and M in the figure indicate the TEDM and TMDM of the molecule, respectively.

**Figure 12:**
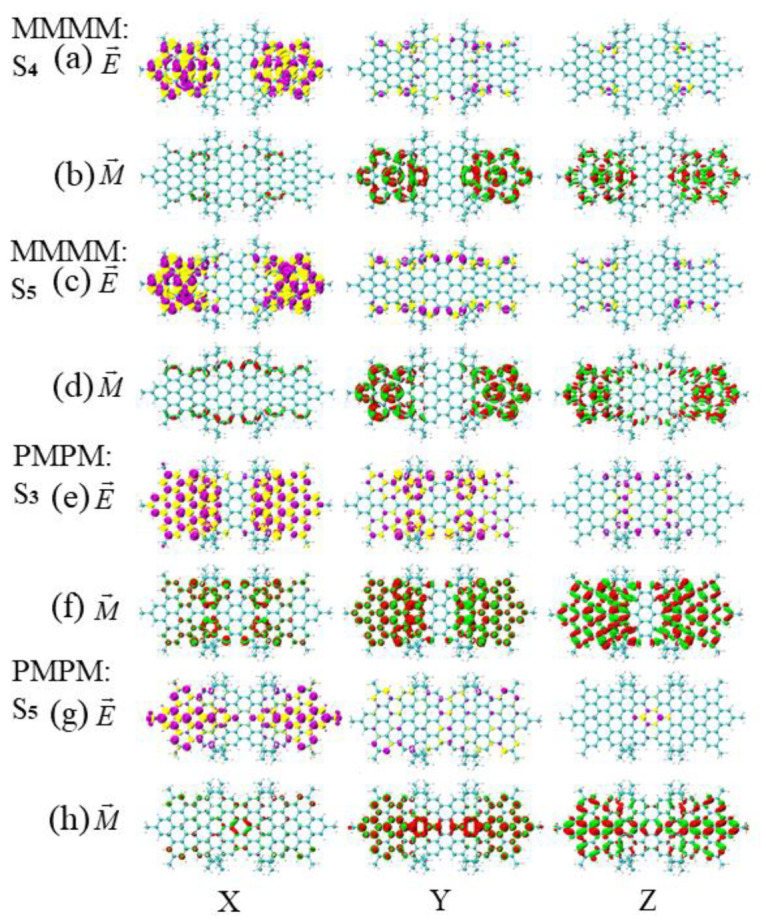
The TEDM and TMDM at similar wavelengths for asymmetric isomers (MMMM) and symmetric diastereomers (PMPM). (**a**,**b**) are distributed as the TEDM and TMDM in the x/y/z direction of S4 of MMMM near the wavelength of 408 nm; (**c**,**d**) are distributed as the TEDM and TMDM in the x/y/z direction of S5 of MMMM near the wavelength of 376 nm; (**e**,**f**) are distributed as the TEDM and TMDM in the x/y/z direction of PMPM near the wavelength of 407 nm; (**g**,**h**) are distributed as the TEDM and TMDM in the x/y/z direction of S5 of PPMM molecules near the wavelength of 376 nm. The letters E and M in the figure indicate the TEDM and TMDM of the molecule, respectively.

The ECD of three new ribbon PAHs isomeric molecules is shown in Figure 7. First, it can be found that the ECD of the two symmetric diastereomers (PMPM and PPMM) is much lower than that of the asymmetric isomer (MMMM), in which the symmetrical diastereomer PMPM is the most obvious, with a difference of almost 50 times. This indicates that the overall degree of distortion of the molecule becomes smaller as the degree of symmetry enhances, which reduces the asymmetric response of TMDM and TEDM in the new ribbon PAHs molecule. Second, the three different isomers show different circular dichroism with different degrees of distortion. Different excited states in the same molecule show different hand shapes, as shown in Figure 7a. The asymmetric isomer (MMMM) has opposite ECD peaks at 332 nm, 344 nm and 353 nm, corresponding to S17, S12 and S11, respectively, as shown in Figure 8. The symmetric diastereomer (PMPM) at 333 nm, 339 nm and 353 nm have opposite ECD peaks, corresponding to S13, S11 and S7, respectively, as shown in Figure 9. The symmetrical diastereomers (PMPM) have opposite ECD peaks at 339 nm, 346 nm, and 354 nm, corresponding to S13, S12, and S11, respectively, as shown in Figure 10. In addition, at around the same wavelength, as shown in Figure 7b, the two symmetric diastereomers (PMPM and PPMM) have their chirality shifted compared to the asymmetric isomer (MMMM). The asymmetric isomer (MMMM) and the symmetric diastereomer (PPMM) show opposite ECD peaks near 438 nm and 474 nm, corresponding to S3, S1 of MMMM and S2, S1 of PPMM, respectively, as shown in Figure 11. The asymmetric isomer (MMMM) and the symmetric diastereomer (PMPM) show opposite ECD peaks near 376 nm and 408 nm, corresponding to S5 and S4 of MMMM and S5 and S3 of PPMM, respectively, as shown in Figure 12.

### 3.3. Energy-Breaking Chiral Reversal

We performed TEDM and TMDM analysis on different excited states within the same molecule, as shown in Figure 8, Figure 9 and Figure 10. The increase of TEDM is represented by the yellow isosurface in the TEDM graph, and the decrease is represented by purple. The increase of TMDM is represented by the red isosurface in the TMDM graph, and the decrease is represented by green. In Figure 8a,b, the interaction between radio waves and magnetic waves in the X and Y directions is symmetric, and the difference is in the Z direction, in which the density of TMDM is very large and mainly distributed on HBC on both sides of the molecule (the distribution of TEDM is very small). The red isosurface on the right side is more distributed than the left side in the Z-direction TMDM, so the TMDM has a transition from left to right. In Figure 8c–f, the TMDM is symmetrically distributed on both sides in the Z direction, and the different points are the TMDM in the Y direction, which are mainly distributed on the HBC on both sides of the molecule. The red isosurface on the right side is more distributed than the left side, indicating that the TMDM has a transition from left to right. Therefore, compared with S13 and S17 of the asymmetric isomer (MMMM), the polarization change in the Z direction causes the chirality of S11 to reverse.

In Figure 9a–f, the interaction between the X and Y directions and the electromagnetic wave components is symmetrical, and the difference is in the Z direction, where the density of TMDM is very large and plays a major role. In Figure 9b,d,f, the TMDM in the Z direction is mainly distributed on both sides. However, the TMDM of S7 and S11 are distributed in red and green, while the TMDM of S13 is mostly red on the left and green on the right. This shows that the TMDM of S13 undergoes a transition from right to left in the Z direction, but S7 and S11 do not. Therefore, an asymmetric magnetic transition dipole moment occurs in the symmetrical diastereomers (PMPM), which causes the chirality of S13 to reverse relative to that of S7 and S11. In Figure 10a–f, the TEDM in the Z direction plays a major role. In Figure 10b,d,f, the TEDM in the Z direction is mainly distributed on both sides. However, the isosurfaces on both sides of the TMDM of S11 and S13 are asymmetric (S11 has more red isosurfaces on the left than on the right, and S13 has more red isosurfaces on the right than on the left), while the TMDM of S12 has symmetrical isosurfaces on both sides. Therefore, the TMDM of the symmetrical diastereomer (PPMM) undergoes an asymmetric transition in the Z direction, which causes the chirality of S11 and S13 to reverse relative to that of S12. In short, the chirality reversal in different excited states of the same molecule is caused by the asymmetric transition of TMDM in the Z direction. The number of asymmetric transitions of the two symmetrical diastereomers (PMPM and PPMM) increases compared with the asymmetric diastereomer (MMMM), which indicates that symmetry systems are more prone to symmetry breaking and resulting in chirality reversal.

### 3.4. Symmetry-Breaking Chiral Reversal

The TEDM and TMDM of S1 of the asymmetric isomer (MMMM) and S1 of the symmetric diastereomer (PPMM) near the wavelength of 474 nm are shown in Figure 11a,c,e,g. It can be found that the red-green isosurfaces on the upper and lower sides of the long axis of the TMDM in the Y direction are unevenly distributed (the red isosurface is mainly on the upper side of the major axis, and the green isosurface is mainly on the lower side of the major axis). The red-green isosurfaces on the upper and lower sides of S1 of the PPMM are unevenly distributed, while the S1 of the MMMM is distributed alternately on both sides of the molecule. Therefore, the in-face polarization of the TMDM of PPMM changes from bottom to top, causing the chirality to reverse. The TEDM and TMDM of S3 of MMMM and S2 of PPMM near the wavelength of 438 nm are shown in Figure 11b,d,f,h. It can be found that the red-green isosurface distribution of TMDM is different in the Y and Z directions. The S3 of MMMM is mainly distributed on the benzene ring in the direction of the main long axis, while the S2 of PPMM is mainly distributed on the HBC on both sides of the molecule. Thus, the polarization region of TMDM is different, causing the chirality of PPMM to reverse.

The TEDM and TMDM of S4 of the asymmetric isomer (MMMM) and S3 of the symmetric diastereomer (PMPM) near the wavelength of 408 nm are shown in Figure 12a,c,e,g. It can be found that the TMDM in the Y direction is significantly different. Among them, the red-green isosurfaces of S4 of MMMM are distributed alternately on both HBCs, while the red-green isosurfaces of S3 of PPMM are distributed on the left and right sides (the red isosurfaces are mainly on the left, and the green isosurfaces are mainly on the right). Thus, the in-plane polarization direction of TMDM changes, causing the chirality of PMPM to reverse. The TEDM and TMDM of S5 of MMMM and S5 of PMPM near the wavelength of 376 nm are shown in Figure 12 b,d,f,h. It can be found that the red-green isosurface distributions of TMDM in the Y and Z directions are different. Among them, the S5 of MMMM is mainly distributed on the HBC of both sides, and the S5 of PPMM is mainly distributed on the benzene ring in the main long axis direction, so the different polarization regions of TMDM promote the reversal of the chirality of PMPM.

According to the energy wavelength formula E=hc/λ, if the wavelength is fixed, then the energy is fixed. In the vicinity of the same wavelength, the excited states of the three isomeric molecules are similar in energy. The in-plane polarization of TMDM in PMPM and PPMM changes compared with MMM, resulting in chirality inversion. The in-plane polarization direction of S1 of PPMM and S3 of PMPM changes in the Y direction TMDM, and the in-plane polarization area S2 of PPMM and S5 of PMPM changes in the Y direction TMDM.

## 4. Conclusions

The molecule studied in this paper is an optically active diastereomer, which is a new type of ribbon-shaped PAHs molecule with three isomers: asymmetric diastereomers (MMMM) and two symmetrical diastereomers (PMPM and PPMM). Our research found that symmetry caused the molecular structure to be distorted, which promoted changes in the TDM of the excited states of PMPM and PPMM, resulting in a shift in the UV-Vis spectrum. Symmetry reduced the asymmetric response of TEDM and TMDM in the new ribbon PAHs molecule, resulting in a much lower ECD of PMPM and PPMM than MMMM. Symmetry breaking of TMDM in the Z direction under the same molecule and different energy caused chiral reversal, wherein symmetry systems were more prone to symmetry breaking. Symmetry under the same energy changed the in-plane polarization mode of TMDM in the Y direction of PMPM and PPMM, which caused the reversal of chirality relative to MMMM. This paper analyzed, in detail, the physical mechanism of the change of molecular chirality by symmetry breaking and in-plane polarization. Our research provides new ideas and theoretical basis for the design of molecular chirality, which can also help to develop more special and efficient chiral molecules.

## Data Availability

Not available.

## Sample Availability

Samples of the compounds are not available from the authors.
